# Stress, Epigenetics, and Alcoholism

**DOI:** 10.35946/arcr.v34.4.13

**Published:** 2012

**Authors:** Sachin Moonat, Subhash C. Pandey

**Affiliations:** **Sachin Moonat, M.S.,***is a doctoral student in the neuroscience graduate program at the University of Illinois at Chicago, Chicago, Illinois.*; **Subhash C. Pandey, Ph.D.,***is a professor and director of Neuroscience Alcoholism Research in the Department of Psychiatry and Department of Anatomy and Cell Biology at the University of Illinois at Chicago and a Veterans Affairs career scientist at the Jesse Brown Veterans Affairs Medical Center, both in Chicago, Illinois.*

**Keywords:** Alcoholism, alcohol consumption, genetic factors, epigenetics, acute stressors, anxiety disorders, stress-related psychiatric disorders, physiological response to stress, dysphoria, brain, brain-derived neurotrophic factor (BDNF), synaptic plasticity

## Abstract

Acute and chronic stressors have been associated with alterations in mood and increased anxiety that may eventually result in the development of stress-related psychiatric disorders. Stress and associated disorders, including anxiety, are key factors in the development of alcoholism because alcohol consumption can temporarily reduce the drinker’s dysphoria. One molecule that may help mediate the relationship between stress and alcohol consumption is brain-derived neurotrophic factor (BDNF), a protein that regulates the structure and function of the sites where two nerve cells interact and exchange nerve signals (i.e., synapses) and which is involved in numerous physiological processes. Aberrant regulation of BDNF signaling and alterations in synapse activity (i.e., synaptic plasticity) have been associated with the pathophysiology of stress-related disorders and alcoholism. Mechanisms that contribute to the regulation of genetic information without modification of the DNA sequence (i.e., epigenetic mechanisms) may play a role in the complex control of BDNF signaling and synaptic plasticity—for example, by modifying the structure of the DNA–protein complexes (i.e., chromatin) that make up the chromosomes and thereby modulating the expression of certain genes. Studies regarding the epigenetic control of BDNF signaling and synaptic plasticity provide a promising direction to understand the mechanisms mediating the interaction between stress and alcoholism.

Alcoholism is a complex disorder characterized by compulsive alcohol seeking and consumption that also is impacted by related psychiatric states, such as anxiety (Koob 2003; Pandey 2003). Both environmental and genetic factors influence alcohol drinking patterns and may increase susceptibility to the development of alcohol addiction (Cloninger 1987; Crabbe 2002). The presence or development of comorbid stress-related psychiatric disorders, which typically are characterized by features such as altered mood and anxiety, often has been associated with an increased propensity for alcoholism (Bolton et al. 2009; Grant et al. 2004; Schuckit and Hesselbrock 1994). More specifically, alcohol consumption is thought to reduce negative symptoms such as depressed mood and anxiety (i.e., dysphoria) linked with stress-related disorders, which ultimately results in self-medication (Bolton et al. 2009; Robinson et al. 2009).

Acute and chronic stressors also may be important factors in regulating alcohol craving and may play a significant role in the relapse to alcohol and drug dependence (Breese et al. 2011; Self and Nestler 1998; Sinha 2007; Uhart and Wand 2009). Various forms of stress, including early-life stress; severe acute stress, such as that experienced in posttraumatic stress disorder (PTSD); and chronic stress, likely can be associated with an increased risk of alcohol and drug dependence (Gordon 2002; Sinha 2008; Uhart and Wand 2009). At the same time, early alcohol exposure and acute alcohol withdrawal may increase vulnerability to stress that may result in the development of negative affective states, such as anxiety or depression (Guerri and Pascual 2010; Hellemans et al. 2010; Koob 2003; Pandey 2003). Taken together, these findings delineate an intricate and complex relationship between stress and alcohol exposure and have stimulated various lines of research that attempt to identify the molecular mechanisms involved in the development of dysphoric symptoms related to the pathophysiology of alcoholism (Koob 2003; Moonat et al. 2010; Pandey 2003).

One focus of this research is the hypothalamus, a key brain region involved in the body’s two main stress response systems: (1) the hormonal system known as the hypothalamic–pituitary–adrenal axis that culminates in the release of stress hormones from the adrenal glands to elicit responses throughout the body and (2) the brain’s central stress response system that includes clusters of brain cells (i.e., nuclei) in the limbic system and autonomic centers of the brain stem (Koob 2008; Smith and Vale 2006). The neurocircuitry related to the central stress response comprises connections between various hypothalamic nuclei, the hippocampus, brain stem nuclei, and a system of interconnected nuclei in the basal forebrain, the extended amygdala (Koob 2008, 2009). These include the central nucleus of amygdala (CeA), medial nucleus of amygdala (MeA), bed nucleus of the stria terminalis, and the shell of the nucleus accumbens (NAc) (Alheid 2003; Koob 2003). Some regions of the extended amygdala, such as the CeA, also have been associated with the development of alcoholism and stress-related disorders such as anxiety, suggesting that the extended amygdala is a neuroanatomical substrate for the interaction between stress and alcoholism (Koob and Volkow 2010; Pandey 2003, 2004).

One mechanism that may provide a link between stress-related psychiatric disorders and alcoholism is modification of synaptic plasticity via neuroadaptation (figure 1) (Moonat et al. 2010; Pandey et al. 2008*b*; Pittenger and Duman 2008). Studies found that ethanol exposure and related withdrawal symptoms can result in structural and functional modifications at the sites where two nerve cells (i.e., neurons) interact and transmit nerve signals (i.e., at the synapse). These modifications at the synaptic level have been observed in various brain regions as well as in neuronal cultures (Carpenter-Hyland and Chandler 2006; Pandey et al. 2008*b*; Roberto et al. 2002; Zhou et al. 2007). Chronic stress also is associated with changes in structural and functional plasticity in various brain regions, including the hippocampus, amygdala, and prefrontal cortex (Goldwater et al. 2009; Pavlides et al. 2002; Roozendaal et al. 2009). Neuroadaptation associated with ethanol exposure or stress plays a role in the onset of dysphoric symptoms that may manifest as stress-related psychiatric disorders or withdrawal-induced anxiety (Pandey et al. 2008*b*; Pittenger and Duman 2008; Roozendaal et al. 2009).

One molecule that has been implicated in synaptic plasticity and long-term memory formation is a protein, cAMP-responsive element binding (CREB) (Abel and Kandel 1998; Alberini 2009; Waltereit and Weller 2003). It also has been recognized as a critical modulator of neuroadaptation associated with alcoholism (Misra et al. 2001; Pandey 2004; Pandey et al. 2003, 2005) and the effects of stress (Barrot et al. 2002; Bilang-Bleuel et al. 2002; Carlezon et al. 2005). CREB is a transcription factor—that is, it helps regulate the first step in the conversion of the genetic information encoded in the DNA into finished protein products (i.e., transcription) by binding to specific DNA sequences in its target genes. To exert its effects, CREB must be activated by the addition of a phosphate group to (i.e., phosphorylation of) the amino acid serine at position 133 of the CREB protein. This phosphorylation is performed by enzymes, protein kinases, that are associated with various signaling cascades, including the mitogen-activated protein kinase (MAPK) pathway (Impey et al. 1999; Shaywitz and Greenberg 1999; Waltereit and Weller 2003). One target gene of CREB encodes a molecule, brain-derived neurotrophic factor (BDNF), which plays an important role in the regulation of synaptic plasticity and dendritic spine structure (Minichiello 2009; Poo 2001; Tao et al. 1998; Soule et al. 2006). (For more information on dendritic spines, see the textbox “Histone Acetylation and Dendritic Spines.”) BDNF also may mediate changes in synaptic plasticity that accompany both alcohol exposure (Moonat et al. 2010, 2011; Pandey et al. 2008*b*) and stress (Briand and Blendy 2010; Duman and Monteggia 2006). Accordingly, researchers have begun to investigate how BDNF activity is controlled. These studies have determined that mechanisms contributing to the regulation of gene transcription that do not involve alterations of the DNA sequence (i.e., epigenetic mechanisms) seem to play a role in the regulation of BDNF activity as well as in synaptic plasticity (Guan et al. 2009; Lubin et al. 2008; Tsankova et al. 2006). Accordingly, this topic has become a focus of research in stress and alcoholism (Elliott et al. 2010; Hunter et al. 2009; Moonat et al. 2010; Pandey et al. 2008*a*; Qiang et al. 2010).

This article reviews research that attempts to describe the role of epigenetic mechanisms in the regulation of BDNF function in alcoholism and stress. After providing an overview of epigenetic mechanisms and their role in the control of gene transcription, the article will summarize research regarding the regulation of BDNF signaling, focusing on epigenetic mechanisms involved in the regulation of BDNF expression. Finally, the article will outline the potential role of the epigenetic control of BDNF signaling and synaptic plasticity in alcoholism and stress.

## Epigenetic Regulation of Gene Transcription

The term epigenetics refers to chemical modifications occurring within a genome that may modulate gene expression without changing the DNA sequence (Holliday 2006; Murrell et al. 2005; Waddington 1942). Common epigenetic alterations include the chemical modification (e.g., addition or removal of acetyl groups) of the proteins around which the DNA is wrapped (i.e., histone proteins) to form the chromosomes and the direct addition of methyl groups (i.e., methylation) to DNA. These modifications are performed by enzymes, such as histone deacetylases (HDACs) and DNA methyltransferases (DNMTs). Both of these mechanisms work in concert to remodel the structure of the protein–DNA complex (i.e., the chromatin), thereby regulating the access of the transcriptional machinery to the DNA and, consequently, gene expression in the cell (Borrelli et al. 2008; Jenuwein and Allis 2001; Levenson and Sweatt 2005; for more information, also see Starkman et al. 2012).

### Histone Acetylation

The basic unit of chromatin, a nucleosome, consists of four histone protein subtypes that form an octamer around which the DNA is wrapped (Jenuwein and Allis 2001; Smith 1991). Histone modification occurs at lysine amino acids near one end of the histone proteins and, as mentioned earlier, involves the addition and removal of acetyl groups. The level of acetylation of the histones determines how tightly the DNA is wound around the histones and how tightly the nucleosomes are stacked together. In the presence of many acetyl groups (i.e., hyperacetylation) at specific lysine residue of histones H3 and H4, the chromatin is relaxed and accessible to the transcriptional proteins, resulting in increased gene transcription; conversely, in the presence of only few acetyl groups (i.e., hypoacetylation), the chromatin is condensed, preventing access of transcriptional proteins and resulting in gene silencing (Smith 1991; Strahl and Allis 2000).

**HDAC-Induced Histone Deacetylation and Dendritic Spines**

Dendritic spines are protuberances that make up the sites where incoming signals from other nerve cells are received (i.e., the post-synaptic terminals) along dendritic processes. The overall number of dendritic spines, their shape, and their distribution on the dendritic processes can change rapidly. This compartmentalization of dendritic spines may allow for the regulation of synaptic plasticity at an individual synapse (Yuste 2011; Higley and Sabatini 2008). For example, various intracellular signaling mechanisms, including brain-derived neurotrophic factor (BDNF) signaling via activity-regulated cytoskeleton-associated (Arc) protein, can regulate the structural and functional components of dendritic spines associated with long-term potentiation (LTP) and synaptic plasticity (Bramham et al. 2008; Minichiello 2009; Soule et al. 2006).

Epigenetic mechanisms also may play a role in the regulation of dendritic spines. Specifically, a recent study (Guan et al. 2009) noted that one histone deacetylase (HDAC) subtype, HDAC2, is involved in the regulation of dendritic spines. When studying mice that produced excessive levels of HDAC2, the investigators found that increased HDAC2 levels were associated with reduced memory formation in a fear-conditioning paradigm and that this impairment was associated with a reduction in dendritic spine density in the hippocampus. Treatment of HDAC2-overexpressing mice with HDAC inhibitors reversed these deficits. On the other hand, animals in which the gene encoding HDAC2 was inactivated (i.e., HDAC2 knockout animals) showed improved learning and increased dendritic spine density (Guan et al. 2009). These findings suggest that HDAC2 plays a role in the regulation of synaptic plasticity; however, future studies may be necessary to identify the specific genes that are regulated by HDAC2 in the control of neuronal function and structure. Given the involvement of brain-derived neurotrophic factor (BDNF) in synaptic plasticity, it may be useful to evaluate the potential regulation of BDNF signaling by HDAC2 in learning at the neuronal and behavioral levels.

**Figure 2:**
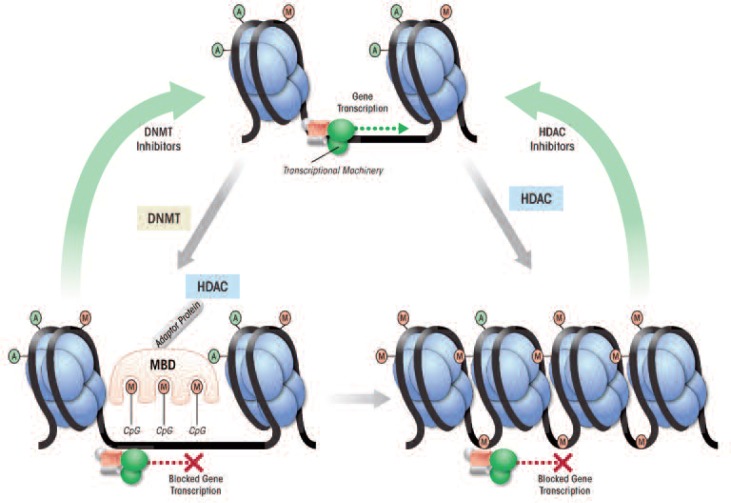
The coordinated actions of proteins involved in epigenetic modifications that regulate gene transcriptional processes. During the 3rst step in the conversion of genetic information encoded in the DNA into gene products (i.e., during gene transcription), the DNA to be transcribed is associated with histone proteins (light blue) that are modified by the addition of acetyl groups (green). This modification results in a relaxed chromatin configuration that allows the transcriptional machinery access to the DNA. Enzymes, DNA methyltransferases (DNMTs), can add methyl groups (red) to the DNA at certain sequences of DNA building blocks (i.e., CpG islands). This methylation causes recruitment of methyl binding domain (MBD) protein complexes that also include repressor proteins, such as histone deacetylases (HDAC). The HDACs remove acetyl groups from histone proteins, resulting in a condensed chromatin that limits the binding of the transcriptional machinery, thereby decreasing gene transcription. Thus, activation of both DNMT and HDAC causes a reduction in gene transcription. Treatment with DNMT inhibitors and HDAC inhibitors may block these enzymatic processes and return the chromatin to a relaxed state, allowing gene transcription.

HDACs are enzymes that can remove acetyl groups from histone proteins; they seem to be key elements in the regulation of chromatin structure and function (figure 2) (Jenuwein and Allis 2001). Inhibition of HDAC enzymes by pharmacological intervention is effective in the treatment of some have been approved or are in clinical trials for this purpose (Dokmanovic et al. 2007; Lane and Chabner 2009). Recently, HDAC inhibitors also have been explored as potential therapeutic agents in the treatment of psychiatric disorders, including stress-related disorders and addiction, and have become an important focus of research in the neuroscience field (Covington et al. 2009; Kumar et al. 2005; Pandey et al. 2008*a*; Renthal and Nestler 2008; Tsankova et al. 2007). Several HDAC isoforms have been identified and grouped into four classes based upon their regulation and cellular localization (de Ruijter et al. 2003; Dokmanovic et al. 2007). Specific HDAC variants (i.e., isoforms) recently have been identified as regulators of neuronal processes such as synaptic plasticity (Guan et al. 2009; Renthal and Nestler 2008). This suggests that use of isoform-specific HDAC inhibitors may increase the specificity and efficacy of these drugs in the treatment of psychiatric disorders.

### DNA Methylation

The chromatin structure also can be modified by adding methyl groups to certain DNA building blocks (i.e., cytosine nucleotides) in a particular gene, resulting in transcriptional silencing (see figure 2). The level of DNA methylation is controlled by three DNMT subtypes that seem to be differentially regulated and preferentially methylate at specific DNA sequences (Antequera 2003; Bestor 2000; Okano et al. 1999). DNA methylation can inhibit transcription either directly, by blocking the binding of transcriptional machinery to DNA, or indirectly, via methyl-CpG binding domain proteins (MBDs) (Fan and Hutnick 2005; Wade 2001).

These proteins, including MeCP2, seem to directly regulate the condensation of chromatin structure and recruit HDACs and DNMTs, which may further enzymatically modify chromatin components (see figure 2) (Fuks et al. 2000; Kimura and Shiota 2003; Nan et al. 1998). Mutations in the *MeCP2* gene and, consequently, the resulting protein that alter transcription of the gene encoding BDNF and affect synaptic plasticity are thought to underlie a neurodevelopmental disorder, Rett syndrome (Chahrour and Zoghbi 2007; Chang et al. 2006; Monteggia and Kavalali 2009; Zhou et al. 2006). Thus, the coordinated actions of HDACs, DNMTs, and MBDs form a complex regulatory network that modulates neuronal function, and dysregulation of these proteins has been implicated in a variety of psychiatric disorders.

Researchers are beginning to identify the role of epigenetic mechanisms in the regulation of gene transcription related to alcohol exposure and the development of alcoholism (Kim and Shukla 2006; Moonat et al. 2010; Pandey et al. 2008*a*; Qiang et al. 2010). Moreover, histone modifications and DNA methylation are involved in the dysphoric states induced by acute and chronic stress (Elliott et al. 2010; Fuchikami et al. 2009; Hunter et al. 2009; Tsankova et al. 2006). Specifically, various studies have demonstrated that epigenetic mechanisms are involved in the regulation of *BDNF* gene transcription, which in turn plays a role in the modulation of synaptic structure and function (He et al. 2010; Lubin et al. 2008; Tsankova et al. 2006). This will be discussed in the following section.

## The Regulation of BDNF Expression and Signaling

BDNF signaling seems to be an important factor in the intracellular processes which occur following neuronal activation (i.e., activity-dependent processes) that play a role in synaptic plasticity and the regulation of dendritic morphology (Messaoudi et al. 2007; Poo 2001; Soule et al. 2006; Ying et al. 2002). BDNF acts by binding to a receptor molecule, tyrosine kinase B (TrkB), which can phosphorylate other proteins as well as itself. The interaction of TrkB with BDNF results in dimerization and autophosphorylation of the receptor (Minichiello 2009; Reichardt 2006). When the TrkB receptor becomes phosphorylated, it can bind to “adaptor molecules” that then can initiate three primary intracellular signaling cascades (Impey et al. 1999; Minichiello 2009; Reichardt 2006):

- The MAPK pathway;
- The phospatidylinositol 3-kinase (PI3K) pathway; and
- The phospholipase Cγ (PLCγ) pathway.

**Figure 3:**
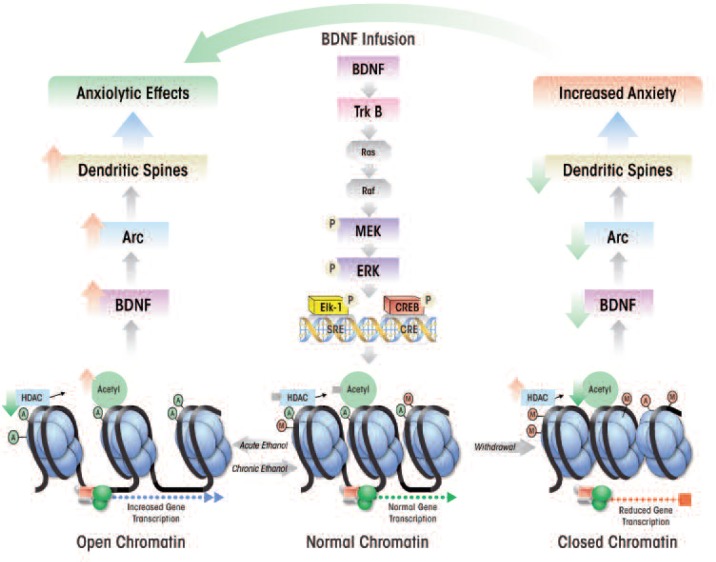
A hypothetical model for the role of brain-derived neurotrophic factor (BDNF) signaling and chromatin remodeling in central amygdaloid brain regions in the regulation of anxiety induced by acute ethanol and ethanol withdrawal. BDNF binding to tyrosine receptor kinase B (TrkB) triggers several signaling cascades that culminate in the activation of transcription factors, Elk-1 and cAMP-responsive element binding protein (CREB). Under normal conditions, histone deacetylase (HDAC) levels and histone acetylation are adequate to allow for normally regulated chromatin structure and gene transcription. Acute ethanol exposure inhibits HDAC, resulting in increased histone acetylation and an open chromatin conformation. This may lead to increased transcription of BDNF as well as higher levels of a protein, activity-regulated cytoskeleton associated protein (Arc), thereby increasing dendritic spine density. The modulation of these synaptic factors results in anxiety-reducing (i.e., anxiolytic) behavioral effects. In contrast during withdrawal from chronic ethanol exposure HDAC activity increases, resulting in a reduction of histone acetylation that in turn closes the chromatin conformation and reduces gene transcription. The resulting low BDNF levels decrease Arc and dendritic spine density, all of which are associated with anxiety-like behaviors. This model is further supported by the fact that exogenous infusion of BDNF into the CeA reduces anxiety-like behaviors in ethanol withdrawn rats and is associated with increased BDNF and Arc levels (Moonat et al. 2010; Pandey et al. 2008*a*, 2008*b*).

The activation of these cascades, particularly the MAPK pathway, ultimately results in the recruitment and phosphorylation of two transcription factors, CREB and Elk-1, which in turn enhance the expression of a gene, *activity-regulated cytoskeleton-associated* (*Arc*) immediate-early gene,^1^ (see figure 3) (Bramham et al. 2008; Pandey et al. 2008*b*; Ramanan et al. 2005; Ying et al. 2002). Arc protein plays a role in the induction of a process, long-term potentiation, and is believed to result in the proliferation of dendritic spines (Huang et al. 2007; Messaoudi et al. 2007; Pandey et al. 2008*b*; Ying et al. 2002). Thus, BDNF plays an important role in the regulation of synaptic plasticity by activating TrkB-coupled signaling and causing induction of *Arc* immediate-early gene.

BDNF is a member of the neurotrophin family whose activity is governed by complex regulatory mechanisms at the transcriptional, translational, and posttranslational levels of gene expression.^2^ The gene encoding BDNF has a complex structure that allows for dynamic control over the expression of the gene region that encodes the actual BDNF protein by allowing for differential regulation of transcription via a wide variety of signaling and epigenetic mechanisms (Aid et al. 2007; Tao et al. 1998; Tsankova et al. 2004). For example, several regulatory elements (i.e., promoters) control *BDNF* transcription, with certain promoters active only in certain cells. As a result, several distinct *BDNF* transcripts (i.e., messenger RNAs [mRNAs]) can be generated that differ in the tissues and cells where they are produced; for example, certain *BDNF* mRNAs specifically are targeted to the neuronal dendrites (Aid et al. 2007; An et al. 2008; Greenberg et al. 2009; Timmusk et al. 1993). Specific *BDNF* transcripts also seem to be differentially regulated by activity-dependent processes. For example, some *BDNF* transcripts are regulated by the CREB transcription factor, and transcription of the same *BDNF* mRNAs is increased after consolidation of fear learning (Lubin et al. 2008; Ou and Gean 2007; Tao et al. 1998). In this manner, BDNF expression is regulated by CREB and, in turn, BDNF signaling also helps modulate CREB activity (Pandey et al. 2008*b*; Pizzorusso et al. 2000; Ying et al. 2002).

### Role of Epigenetic Mechanisms

Epigenetic mechanisms, specifically histone modifications and DNA methylation, regulate BDNF expression via specific promoter regions for the *BDNF* gene. Huang and colleagues (2002) demonstrated that histone acetylation resulted in enhanced BDNF expression. Specifically, the level of histone acetylation associated with *BDNF* promoter II was increased in the hippocampus, suggesting a role for chromatin remodeling in the regulation of *BDNF*. Tsankova and colleagues (2004) also showed that histone acetylation influenced hippocampal BDNF expression in a model of electroconvulsive shock therapy, demonstrating that time-and promoter-dependent changes in histone acetylation levels were associated with similar changes in BDNF expression. Other investigators subsequently found that histone modifications were involved in the regulation of BDNF expression in the striatum during chronic cocaine exposure and in the hippocampus in a model of depression induced by chronic social-defeat stress (Kumar et al. 2005; Tsankova et al. 2006). Importantly, these studies determined that specific HDAC isoforms participated in the complex process of chromatin remodeling, suggesting a therapeutic role for isoform-specific HDAC inhibitors in alcohol and drugs of abuse as well as in depression (Kumar et al. 2005; Tsankova et al. 2006; Renthal and Nestler 2008). (Another role for HDAC activity—namely, in the regulation of dendritic spines—is discussed in the textbox “Histone Deacetylation and Dendritic Spines.”)

As mentioned earlier, DNA methylation can inhibit transcription indirectly, via MBDs that seem to regulate the condensation of chromatin structure and recruit HDACs and DNMTs. One of these MBDs is MeCP2, which represses gene transcription via coordinated binding of methylated DNA, HDACs, and DNMT1 (Ballestar and Wolffe 2001). MeCP2 plays a role in the activity-dependent regulation of BDNF expression in neurons. Specifically, enhanced expression of one of the BDNF variants (i.e., BDNF exon IV) following arrival of a nerve impulse in the neurons (i.e., following depolarization) was associated with increased histone acetylation, reduced DNA methylation, and reduced MeCP2 binding at the promoter for that BDNF variant. This suggests that BDNF expression is regulated dynamically by chromatin remodeling (Martinowich et al. 2003). MeCP2-dependent regulation of this BDNF variant also is involved in regulating the formation of dendritic spines (Zhou et al. 2006).

The association between MeCP2 and BDNF exon IV levels is mediated at least in part by a protein, RACK1. This protein associates with histones H3 and H4 at the *BDNF* exon IV promoter and causes MeCP2 to dissociate from the *BDNF* gene (He et al. 2010). RACK1-mediated dissociation of MeCP2 from the *BDNF* gene leads to increased histone acetylation at the *BDNF* exon IV promoter and, in turn, increases BDNF expression (He et al. 2010). Other studies found that reduction of DNA methylation levels in the *BDNF* exon IV promoter region increased BDNF expression during a fear conditioning experiment (Lubin et al. 2008). Of interest, in that study BDNF exon IV expression specifically was associated with the consolidation of fear memory, whereas increases in other BDNF variants (i.e., BDNF exons I and VI) occurred with the presentation of context alone (Lubin et al. 2008).

Taken together, these findings provide evidence for the overlap between histone modifications and DNA methylation in the regulation of *BDNF* gene expression, which may be associated with activity-dependent changes in synaptic plasticity.

## BDNF and Epigenetic Mechanisms in Stress and Alcoholism

### BDNF and Stress

Chronic stress has been linked with shrinkage of brain tissue (i.e., neuronal atrophy) and modulation of dendritic structure in the hippocampus (McEwen 2008; Watanabe et al. 1992) and was associated with reduced BDNF levels in that brain structure (Smith et al. 1995). In addition, both acute and chronic stress may modulate BDNF levels and structural plasticity in a variety of brain areas, including the hippocampus, prefrontal cortex, and amygdala (Calabrese et al. 2009; McEwen 2008; Pizarro et al. 2004). In the hippocampus, acute stress caused by immobilization as well as swim stress increased the levels of *BDNF* mRNA. This increase was associated with increased MeCP2 phosphorylation, suggesting that epigenetic mechanisms help mediate the effects of acute stress (Marmigere et al. 2003; Molteni et al. 2009). Increased BDNF expression may represent a protective mechanism in response to stress; conversely, reduced BDNF levels after exposure to repetitive and chronic stress appear to represent a dysregulation of this mechanism (Calabrese et al. 2009; McEwen 2008). This assumption is supported by findings that the antidepressant effects of medications used in chronic-stress models of depression are mediated by an increase in BDNF levels in the hippocampus (Nibuya et al. 1995; Shirayama et al. 2002; Tsankova et al. 2006). It also is interesting to note that low BDNF levels in the CeA and MeA mediate anxiety-like behaviors, and the anxiety-reducing (i.e., anxiolytic) effects of alcohol may be associated with an increase in BDNF signaling (Moonat et al. 2011; Pandey et al. 2006, 2008*b*). These observations clearly suggest that aberrations of BDNF signaling contribute to the development of stress-related dysphoric behaviors, and the BDNF signaling pathway therefore may be a promising potential therapeutic target for treatment of these disorders.

#### Role of Chromatin Remodeling

Researchers recently also have begun to investigate the role of chromatin remodeling in BDNF signaling associated with stress-related dysphoria. Using a model of depression induced by chronic stress, Tsankova and colleagues (2006) found that the levels of the *BDNF* exon IV and exon VI were reduced in the hippocampus and that this effect could be blocked by chronic antidepressant treatment (Tsankova et al. 2006). Further analyses found that this effect likely was associated with changes in histone methylation because chronic stress increased the levels of methylated histone H3 protein near the *BDNF* exons IV and VI promoters, which interferes with *BDNF* transcription. Conversely, treatment with antidepressants reduced the levels of histone methylation and increased the levels of acetylated H3 associated with these *BDNF* promoters, thereby increasing BDNF expression. Simultaneously, antidepressant treatment reduced the expression of HDAC5, but when the levels of HDAC5 were elevated through genetic engineering, the effects of antidepressant treatment were reduced (Tsankova et al. 2006).

The levels of several HDACs in the NAc also may influence the development of stress-related dysphoria. In contrast to the hippocampus, HDAC2 and HDAC5 levels in the NAc were reduced by chronic stress, suggesting opposing roles for histone modifications in the hippocampus and NAc in stress-related dysphoria (Renthal et al. 2007). Interestingly, systemic treatment with HDAC inhibitors or infusion of HDAC inhibitors into the NAc reduced stress-related dysphoria (Covington et al. 2009; Tsankova et al. 2006). Taken together, all these results suggest that histone modifications in stress-related dysphoria and the therapeutic effects of antidepressants.

#### Role of DNA Methylation

DNA methylation also plays a role in the development of stress-related dysphoria as well as synaptic plasticity in the NAc. Specifically, chronic stress increased expression of one DNA methyltransferase, DNMT3a, in the NAc, which was associated with an increase in depressive-like behavior (LaPlant et al. 2010). Infusion of a DNMT inhibitor into the NAc of chronically stressed animals reduced these observed behaviors. Conversely, overexpression of DNMT3a in the NAc precipitated a depression-like phenotype in animals that had not been exposed to stress. DNMT3a overexpression also resulted in the proliferation of dendritic spines (LaPlant et al. 2010). These results indicate that DNMT3a may contribute to stress-related dysphoria and control of dendritic spine structure. It would be interesting to expand upon these results and determine if a link exists between stress-associated changes in DNMT3a and methylation of the *BDNF* gene and alcoholism.

### BDNF, Stress, and Alcoholism

Various researchers have explored the association of BDNF with ethanol preference, the effects of ethanol exposure, and dysphoric states associated with withdrawal from chronic ethanol exposure. BDNF deficits may lead to an increased preference for ethanol, because transgenic animals with reduced BDNF expression have a higher ethanol preference and conditioned place preference for ethanol compared with wild-type control animals (Hensler et al. 2003; McGough et al. 2004). Furthermore, ethanol exposure results in increased BDNF expression in the dorsal striatum. This increase involved a regulatory mechanism mediated by RACK1 because exogenous increases in RACK1 led to increased BDNF expression, resulting in reduced ethanol consumption (McGough et al. 2004). These findings suggest that BDNF in the dorsal striatum helps regulate neuronal homeostasis and prevent alcohol addiction (McGough et al. 2004). In addition, endogenous BDNF signaling in the dorsolateral striatum participates in the regulation of ethanol intake (Jeanblanc et al. 2009). Because, as mentioned earlier, MeCP2 is involved in the RACK1-mediated regulation of BDNF (He et al. 2010), future studies should determine whether chromatin remodeling affects BDNF expression in the dorsal striatum and, ultimately, ethanol’s effects and ethanol preference.

Various studies have examined how BDNF impacts the interaction between alcohol preference and anxiety. For example, Pandey and colleagues (2006) reduced BDNF levels in the extended amygdala by introducing small molecules that can inhibit BDNF expression (i.e., antisense oligodeoxynucleotides) into the CeA or MeA. This caused increased voluntary ethanol intake and anxiety-like behaviors. The low BDNF levels resulted in reduced BDNF signaling, as evidenced by decreased levels of the phosphorylated forms of CREB and another regulatory molecule (Pandey et al. 2006). Both the effects on behavior and protein phosphorylation were reversed when BDNF was introduced together with the antisense oligonucleotides (Pandey et al. 2006). Additional studies identified a subsequent step in the signaling cascade induced by BDNF involving the Arc protein mentioned earlier. The findings suggested that the effects of reduced amygdaloid BDNF expression on ethanol preference and anxiety-like behaviors may be mediated by the downstream regulation of Arc (Pandey et al. 2008*b*). These behavioral changes were accompanied by a reduction in dendritic spine density in the CeA.

**Figure 4:**
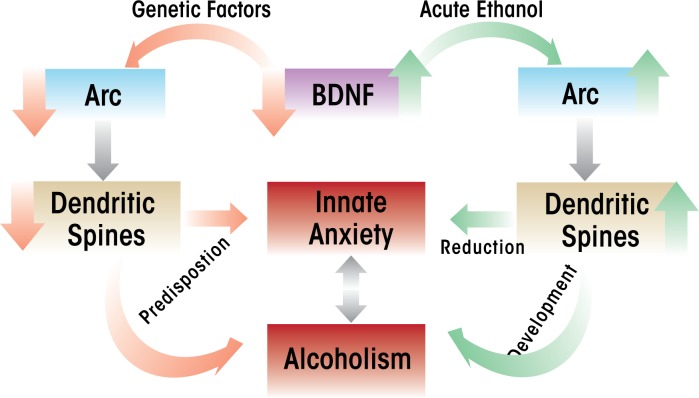
A hypothetical model for the role of amygdaloid brain-derived neurotrophic factor (BDNF) in the regulation of activity-regulated cytoskeleton-associated protein (Arc) and dendritic spine density in the comorbidity between innate anxiety and alcohol preference. Genetic factors may lead to innately low levels of amygdaloid BDNF that result in reduced Arc and dendritic spine density and which are associated with a predisposition to innate anxiety-like behaviors. Acute ethanol exposure increases BDNF signaling and associated synaptic factors, Arc, and dendritic spine density and results in a reduction of innate anxiety. Taken together, innate anxiety and a reduction of this anxiety by acute ethanol may be responsible for the development of alcoholism (Moonat et al. 2011).

In an extension of these findings, investigators used an animal model of genetic predisposition to alcoholism and anxiety (i.e., selectively-bred alcohol-preferring [P] and nonpreferring [NP] rats) to study the role of BDNF in the extended amygdala. The studies found that compared with NP rats, P rats expressed lower levels of BDNF and Arc and had lower dendritic spine density in the CeA and MeA and that these characteristics were associated with high innate anxiety-like behaviors (Moonat et al. 2011; Prakash et al. 2008). Furthermore, acute ethanol exposure had anxiolytic effects that were associated with increased BDNF and Arc levels as well as increased dendritic spine density in the CeA and MeA in P rats, but not in NP rats (Moonat et al. 2011). These findings were consistent with earlier findings in Sprague-Dawley rats, which showed that increases in BDNF–Arc signaling and dendritic spine density in the extended amygdala were associated with the anxiolytic effects of acute ethanol (Pandey et al. 2008*b*). Withdrawal from chronic ethanol exposure provoked anxiety-like behaviors, which resulted in reduced BDNF signaling in the CeA and MeA, whereas BDNF infusion into the CeA normalized Arc levels and prevented anxiety-like behaviors (Pandey et al. 2008*b*). Taken together, these studies suggest that reduced BDNF–Arc signaling and synaptic plasticity contribute to both dysphoria associated with a genetic vulnerability for anxiety and to anxiety induced by environmental stressors, such as alcohol withdrawal (see figures 3 and 4).

Recent findings further suggest that the anxiolytic effects of acute ethanol exposure are associated with reduced HDAC activity and increased histone acetylation in the CeA and MeA (Pandey et al. 2008*a*). Conversely, withdrawal-induced anxiety following chronic ethanol treatment was linked with increased HDAC activity levels and reduced histone acetylation in these amygdaloid brain regions (see figure 3). Systemic administration of an agent that inhibits HDAC activity (i.e., trichostatin A) reduced the effects of withdrawal on histone acetylation and anxiety-like behaviors (Pandey et al. 2008*a*). Thus, treatment with HDAC inhibitors appears to have similar effects on withdrawal-induced anxiety as BDNF, and acute ethanol exposure may have similar effects on histone acetylation and BDNF levels (Pandey et al. 2008*a*, 2008*b*). Therefore, it may be important to study the potential regulation of amygdaloid BDNF by chromatin remodeling and its role in dysphoria associated with the development of alcoholism. Similarly, it may be interesting to explore the possibility that innate abnormalities in chromatin structure may affect BDNF levels, resulting in innate anxiety-like behaviors, such as those demonstrated by P rats, that may be critical to the development of alcoholism.

## Conclusions

The studies reviewed here suggest that the reduction of BDNF levels may play a role in the neuroadaptation to repetitive or chronic exposure to alcohol or stress and the development of dysphoric states. Moreover, it appears that abnormalities in BDNF signaling serve as predisposing factors to innate dysphoric states that may associated with alcohol-drinking behaviors, such as anxiety (see figure 4). It also is possible that the environmental effects and genetic factors involved in an increased vulnerability to stress and alcoholism may be related to a common epigenetic mechanism that results in the dysregulation of BDNF signaling in various brain regions. Future studies are necessary to further evaluate the role of specific HDAC and DNMT variants that are involved in the epigenetic regulation of BDNF or other genes associated with synaptic plasticity during the development of pathological behaviors associated with stress and alcohol addiction. Finally, the development and assessment of specific pharmacological agents that act via epigenetic mechanisms, such as HDAC and DNMT inhibitors, could have a significant psychotherapeutic impact on the development of stress-related disorders and the comorbidity with alcoholism.

## Figures and Tables

**Figure 1:**
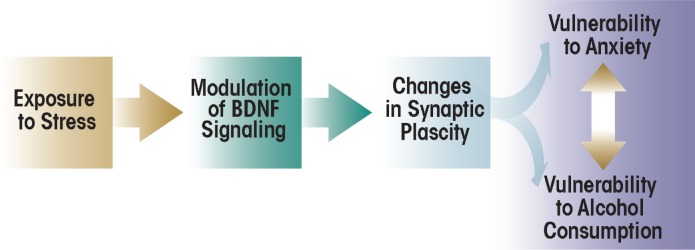
A psychiatric model for the relationship between stress, anxiety, and alcohol consumption and its modulation by brain-derived neurotrophic factor (BDNF) and synaptic plasticity. Exposure to stress is thought to result in the modulation of BDNF and synaptic plasticity in various brain regions. These changes may result in increased vulnerability to the development of stress-related disorders such as anxiety. Vulnerability to alcohol consumption also may be increased, either directly due to stress exposure or in response to the development of anxiety.

## Footnotes

1 This gene also is known as activity-regulated gene 3.1 (Arg3.1).

2 Transcription is the 3rst step of gene expression, in which the genetic information encoded in the DNA is copied into an intermediate molecule, mRNA. In the second step of gene expression, translation, the mRNA then serves as a template for the synthesis of the proteins that are the gene products. After translation (i.e., posttranslationally) these proteins can be modified further by the addition of certain chemical groups.
